# RP-HPLC Characterization of Lupenone and β-Sitosterol in Rhizoma Musae and Evaluation of the Anti-Diabetic Activity of Lupenone in Diabetic Sprague-Dawley Rats

**DOI:** 10.3390/molecules190914114

**Published:** 2014-09-09

**Authors:** Feng Xu, Hongmei Wu, Xiangpei Wang, Ye Yang, Yuanmin Wang, Haibing Qian, Yanyan Zhang

**Affiliations:** Department of Pharmacognosy, Guiyang College of Traditional Chinese Medicine, 50, Nanming District, Guiyang 550002, Guizhou, China; E-Mails: xf333666999@sina.com (F.X.); whm0425@126.com (H.W.); yangye459@163.com (Y.Y.); wym01234@sina.com (Y.W.); qianhaibing888@163.com (H.Q.); zhangyanyan111@sina.com (Y.Z.)

**Keywords:** Rhizoma Musae, diabetes, lupenone, β-sitosterol, HbA1c, cultivation and harvesting

## Abstract

With the aim of characterizing the active ingredients lupenone and β-sitosterol in Rhizoma Musae samples a reversed-phase HPLC method for the separation of these two compounds in Rhizoma Musae samples was developed (regression coefficient > 0.9996). The method was further applied to quantify lupenone and β-sitosterol content in Rhizoma Musae samples cultured in different growth environments. Different variables such as geographical location, growth stage, and harvest time, demonstrated differential effects on lupenone and β-sitosterol levels. Moreover, we determined the optimum conditions for cultivation and harvesting of Rhizoma Musae herbs. Lupenone administration caused a significant reduction in fasting blood glucose (FBG) levels in diabetic rats at doses of 1.78, 5.33, and 16.00 mg·kg^−1^·day^−1^ for 14 days, the glycated hemoglobin (HbA1c) levels of diabetic rats also significantly reduced at doses of 5.33, and 16.00 mg·kg^−1^·day^−1^, indicating a robust antidiabetic activity. To our knowledge, this is the first report of an optimized HPLC method successfully applied to quantify lupenone and β-sitosterol, and its applicability in optimizing Rhizoma Musae growth. Animal experiments also showed for the first time that lupenone from Rhizoma Musae has anti-diabetic activity.

## 1. Introduction

*Musa basjoo* Sieb. et Zucc. (called “*ba-jiao*” in Chinese) is consumed as both food and medicine in various regions of China. The dried rhizome of *ba-jiao* is Rhizoma Musae, recorded in the Compendium of Materia Medica [1]. The herb has been used for centuries in Miao medicine practice for heat-clearing and detoxifying treatments, as well as quenching of thirst and as a diuretic [2,3]. Recent studies have reported that Rhizoma Musae has anti-bacterial, anti–α-glucosidase, anti-diabetic, anti-inflammatory, and analgesic properties [4,5,6,7,8]. In China, Rhizoma Musae is used as a raw material for many pharmaceutical preparations, such as *gukangjiaonang*, *gukangpian*, and *zhongtongshupenwuji*.

**Figure 1:**
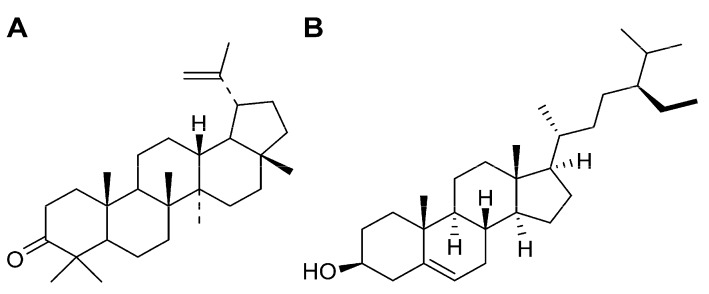
Chemical structure of lupenone (**A**) and β-sitosterol (**B**).

Recently, we successfully isolated lupenone and β-sitosterol (Figure 1) from the ethyl acetate fraction of Rhizoma Musae [9]. Current studies have shown that lupenone and β-sitosterol possess multiple pharmacological activities. For example, *in vitro* studies have found that lupenone stimulates melanogenesis in B_16_ murine melanoma cells [10], as well as inhibits α-glucosidase (α-Glu) and protein tyrosine phosphatase 1B (PTP1B) activities [11,12], Furthermore, lupenone inhibits adipogenic differentiation via downregulation of peroxisome proliferator-activated receptor PPARγ expression in 3T3-L1 cells [13].

Additionally, previous studies have reported that β-sitosterol inhibits the growth of HT-29 human colon cancer cells and induces apoptosis of LNCaP human prostate cancer cells *in vitro* [14,15]. β-Sitosterol also has the potential to function as an anti-cancer agent for controlling colon carcinogenesis [16]. Moreover, β-sitosterol possessed potential anti-diabetic and anti-oxidant activities in streptozotocin-induced hyperglycemia models reported by Gupta *et al.* [17].

Lupenone and β-sitosterol are likely the primary bioactive constituents responsible for the therapeutic efficacies of Rhizoma Musae [11,12,17]. Managing the active ingredients in herbs may be a feasible approach to control the therapeutic qualities of medicinal herbs [18,19]. To the best of our knowledge, no method has been reported for the characterization of lupenone and β-sitosterol in Rhizoma Musae, an important strategy in the comprehensive evaluation of lupenone and β-sitosterol in Rhizoma Musae cultivation.

For the first time, this study monitored and quantified lupenone by HPLC. A simple method was developed for concomitant determination of lupenone and β-sitosterol in Rhizoma Musae based on HPLC-UV. This method can be used for the quality control of Rhizoma Musae and help guide optimal cultivation and harvesting of the herb. This study also characterizes the glucose-lowering effect of lupenone in experimental type-II diabetes.

## 2. Results and Discussion

### 2.1. Optimization of Sample Preparation

The effect of extraction method, solvent and time on the extraction efficiency was investigated. The results are summarized in Table 1. In examining the differences between ultrasonic and reflux extraction of Rhizoma Musae samples, refluxing was observed to completely extract lupenone and β-sitosterol. When comparing each extraction of 1-, 2-, and 3-cycle 1.5 h-long extractions of Rhizoma Musae, it was observed that a 2-cycle extraction of lupenone and β-sitosterol resulted in complete extraction. Methanol, ethanol, 95% ethanol, 50% methanol and 50% ethanol were evaluated as the extraction solvents, and methanol was observed to possess the greatest extraction efficiency. While using the 2-cycle 1.5 h-long extraction of Rhizoma Musae by ultrasound the result showed that the extraction does not occur at exactly the same extraction time. Therefore, the optimum extraction method consisted of methanol refluxing of the extract for two 1.5 h-long cycles.

**Table 1:** Optimization of sample extraction conditions.

Sample Extraction Conditions	Lupenone (µg) ± SD	β-Sitosterol (µg) ± SD
Refluxing (Methanol)	188.1 ± 3.7	297.9 ± 5.7
Ultrasonic extraction (Methanol)	170.5 ± 4.4	294.4 ± 7.0
Methanol refluxing	188.1 ± 3.7	297.9 ± 5.7
Ethanol refluxing	176.2 ± 4.1	282.8 ± 5.2
95% ethanol refluxing	188.1 ± 3.7	282.3 ± 6.4
50% methanol refluxing	154.9 ± 3.9	not detected
50% ethanol refluxing	183.6 ± 3.6	262.27 ± 6.2
Methanol under reflux for 1 time (1.5 h/times)	174.3 ± 4.3	282.3 ± 6.1
Methanol under reflux for 2 times (1.5 h/times)	188.1 ± 3.7	297.9 ± 5.7
Methanol under reflux for 3 times (1.5 h/times)	188.1 ± 3.6	298.1 ± 5.3

### 2.2. Optimization of Chromatographic Conditions

**Figure 2:**
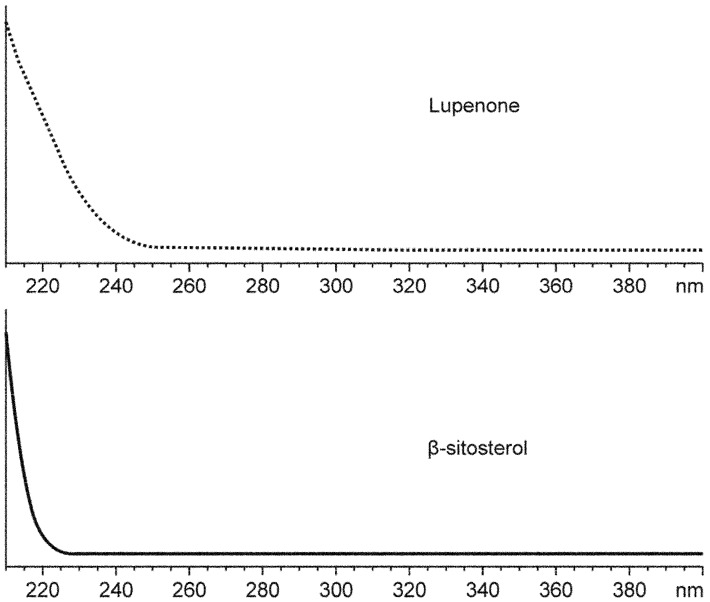
Ultraviolet full-band scan results of lupenone and β-sitosterol.

Full-band UV scanning results showed that the detection signals of lupenone and β-sitosterol were stronger at 206 nm (Figure 2), and that monitoring the absorbance of 206 nm was therefore conducive to the detection of both lupenone and β-sitosterol.

A comparison of the different proportions of mobile phase constituents (methanol, acetonitrile, water, 0.1% acetic acid in water, and 0.1% aqueous phosphoric acid), found that a methanol-acetonitrile (1:1) mixture achieved complete separation. The use of a double-pump caused instability in the baseline, perhaps because, at low wavelengths, the mobile phase was not uniformly mixed in the device, and was accompanied by temperature fluctuations upon mixing. Through the primary configuration and application of a single pump, mixing of the mobile phase in the device can be minimized. 

The temperature of column and flow-rate, were evaluated at different column temperatures ranging from 25 to 35 °C and the flow-rate from 0.5 to 1.0 mL/min. The desired separation of lupenone and β-sitosterol was obtained by using methanol-acetonitrile (1:1) as the mobile phase, with fractions measured at 206 nm wavelength, a column temperature of 25 °C, and a flow rate of 0.7 mL/min.

### 2.3. Method Validation

**Figure 3:**
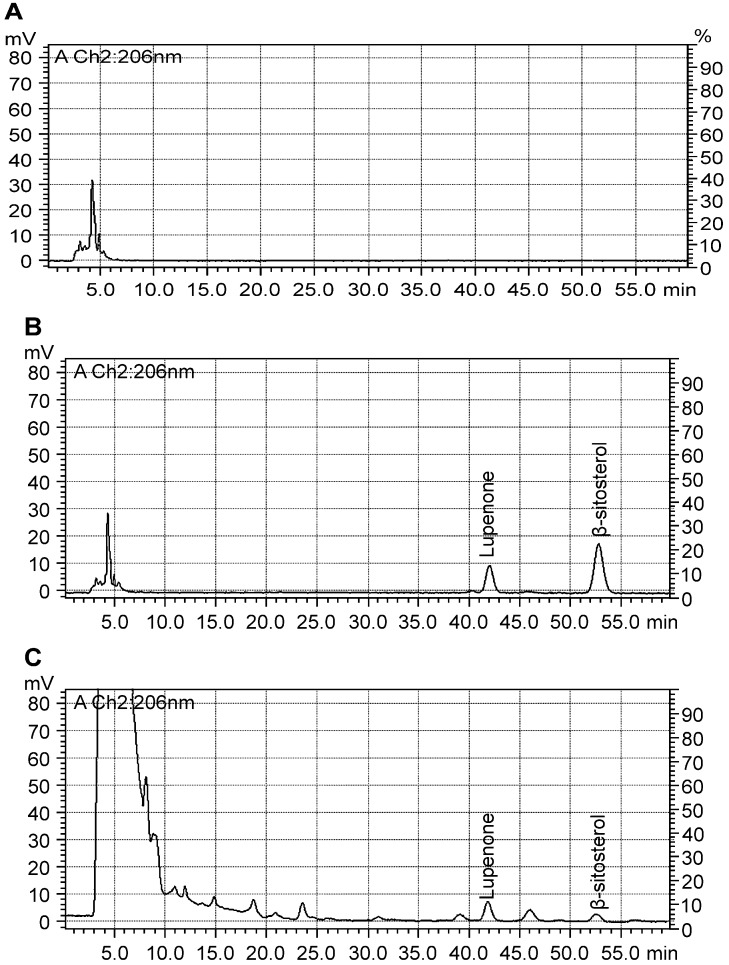
Representative HPLC Chromatograms of Blank (**A**), Standards (**B**) and Rhizoma Musae Samples Collected from Guiyang (**C**).

The results from Table 2 and Table 3 suggest that a superior method for the determination of specificity, precision, accuracy stability and repeatability exists to quantify lupenone and β-sitosterol content in Rhizoma Musae. Method specificity was apparent by the baseline chromatographic separation of the two components (Figure 3) with a resolution factor of greater than 1.5. The correlation coefficients of the calibration curves for lupenone and β-sitosterol were 0.9998 and 0.9996, respectively, indicating a good linear detector response in the investigated dynamic range. The LOD and LOQ for lupenone and β-sitosterol were less than 4.21 µg/mL and 21.52 µg/mL, respectively (Table 2), showing that the analytes have high sensitivity. The RSD of precision was <2.51%, and the recoveries ranged from 96.77% to 104.52%; this indicated that the method allows highly accurate and precise simultaneous analysis of the two analytes. The sample solution can be regarded as stable within 24 h, because the RSD values were <2.24%. The RSD values of the component contents were <2.83, which suggested the repeatability is good.

**Table 2:** Calibration curve values for lupenone and β-sitosterol.

Analytes	Calibration Curves	r	Test Range (µg/mL)	LOD (µg/mL)	LOQ (µg/mL)
Lupenone	y = 7543.5x − 63311	0.9998	50.25–402.00	3.05	14.01
β-sitosterol	y = 5505.8x − 71182	0.9996	50.50–404.00	4.21	21.52

y: Peak area; x: concentration (µg/mL).

**Table 3:** Precision, Repeatability, Stability, and Recovery of lupenone and β-sitosterol.

Analytes	Original (µg)	Spiked (µg)	Found (µg)	Recovery (%)	RSD (%)	Precision		Repeatability RSD (%)	Stability RSD (%)
						Intraday Variability RSD (%)	Interday Variability RSD (%)		
Lupenone	317.3	142.5	455.2	96.77					
Lupenone	317.3	142.5	456.8	97.89	1.71				
Lupenone	317.3	142.5	459.9	100.07					
Lupenone	317.0	285.0	608.9	102.42					
Lupenone	317.0	285.0	610.5	102.98	0.50	0.97	2.51	2.46	2.24
Lupenone	317.0	285.0	611.8	103.44					
Lupenone	317.4	570.0	903.8	102.88					
Lupenone	317.4	570.0	900.8	102.35	0.36				
Lupenone	317.4	570.0	904.8	103.05					
β-sitosterol	352.6	110.5	463.4	100.27					
β-sitosterol	352.6	110.5	460.6	97.74	1.60				
β-sitosterol	352.6	110.5	460.2	97.38					
β-sitosterol	352.0	221.0	580.6	103.44					
β-sitosterol	352.0	221.0	580.7	103.48	0.59	1.33	1.67	2.83	1.53
β-sitosterol	352.0	221.0	583.0	104.52					
β-sitosterol	352.6	442.0	812.1	103.98					
β-sitosterol	352.6	442.0	810.1	103.51	0.91				
β-sitosterol	352.6	442.00	804.2	102.17					

### 2.4. Quantitative Determination of Lupenone and β-Sitosterol in Rhizoma Musae

Thirty seven Rhizoma Musae samples from different altitudes, origins, harvest months, harvest times (within a day), and growth stages were analyzed with the developed quantification method. The contents of lupenone and β-sitosterol varied markedly with each sample (Table 4, Table 5, Table 6 and Table 7).

**Table 4:** Levels of lupenone and β-sitosterol in Rhizoma Musae samples from different origin.

Location (Rhizoma Musae Samples)	Harvest Date (YYYY.M.DD)	Altitude (m)	Lupenone Content (µg/g) ± SD	β-Sitosterol Content (µg/g) ± SD
Tianzhu County, Guizhou Prov. (1)	2012.7.21	298.52	320.4 ± 6.5	675.5 ± 13.3
Tianzhu County, Guizhou Prov. (2)	2012.7.22	300.46	171.2 ± 3.5	251.4 ± 4.8
Jinping County, Guizhou Prov.	2012.7.21	319.35	395.9 ± 4.6	368.0 ± 6.5
Lezhi County, Sichuan Prov.	2012.7.21	350.65	269.6 ± 6.5	254.6 ± 4.5
Zhizhong County, Sichuan Prov.	2012.8.31	413.10	2576.0 ± 60.0	460.7 ± 7.8
Jianhe County, Guizhou Prov.	2012.7.30	541.60	857.3 ± 19.0	257.07 ± 5.8
Jinsha County, Guizhou Prov.	2012.8.19	800.79	800.1 ± 20.1	209.0 ± 4.5
Majiang County, Guizhou Prov.	2012.7.24	891.80	202.1 ± 3.6	262.0 ± 7.2
Longli County, Guizhou Prov.	2012.7.19	1000.56	494.3 ± 5.6	1183.8 ± 16.8
Guiyang city, Guizhou Prov.	2012.7.17	1078.20	783.4 ± 11.9	734.5 ± 13.6
Zhenfeng County, Guizhou Prov.	2012.7.14	1143.58	513.6 ± 12.3	445.0 ± 8.0
Medog County, town Motuo	2012.7.15	1253.92	2123.4 ± 41.5	471.6 ± 11.8
Sanjiang town, Guizhou Prov.	2013.6.25	1357.82	256.5 ± 3.1	602.4 ± 12.5

**Table 5:** Lupenone and β-sitosterol levels at different harvest times of Rhizoma Musae sample collection.

Sample Month	Lupenone Content (µg/g) ± SD	β-Sitosterol Content (µg/g) ± SD
January	209.9 ± 4.3	312.9 ± 6.2
February	534.4 ± 12.1	464.1 ± 7.8
March	261.9 ± 7.3	468.0 ± 8.7
April	278.1 ± 5.2	467.6 ± 5.4
May	498.2 ± 9.1	360.7 ± 9.2
June	1562.5 ± 20.6	542.7 ± 12.7
July	296.6 ± 11.8	299.3 ± 7.1
August	244.9 ± 4.2	359.6 ± 6.8
September	176.7 ± 5.8	357.0 ± 5.4
October	204.2 ± 4.3	290.5 ± 4.1
November	225.8 ± 5.9	255.5 ± 6.6
December	178.9 ± 3.9	214.9 ± 4.3

**Table 6:** Lupenone and β-sitosterol levels in Rhizoma Musae samples collected at different time periods within a single day.

Sample Time (o’clock)	Lupenone Content (µg/g) ± SD	β-Sitosterol Content (µg/g) ± SD
8:00	2586.1 ± 32.1	461.0 ± 7.8
10:00	2041.9 ± 28.9	584.3 ± 15.4
12:00	1022.9 ± 18.3	749.4 ± 12.1
14:00	1824.5 ± 28.2	482.0 ± 12.9
16:00	2485.8 ± 56.0	511.3 ± 12.7
18:00	2326.3 ± 33.3	413.4 ± 7.8

**Table 7:** Levels of lupenone and β-sitosterol in Rhizoma Musae samples at different growth stages.

Samples (cm)	Lupenone Content (µg/g) ± SD	β-Sitosterol Content (µg/g) ± SD
5	212.9 ± 4.4	397.6 ± 7.8
7	363.8 ± 6.2	437.0 ± 7.3
10	406.6 ± 9.3	397.8 ± 8.9
13	362.6 ± 8.0	383.2 ± 7.4
15	253.5 ± 5.9	283.3 ± 7.4
20	186.3 ± 4.5	267.2 ± 4.2

**Figure 4:**
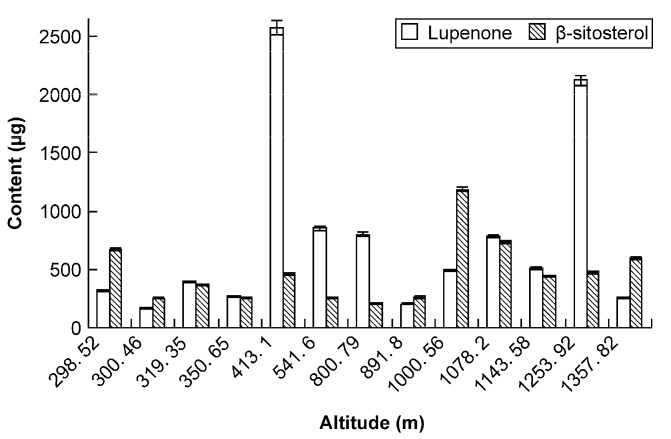
Lupenone and β-sitosterol levels in Rhizoma Musae samples from different altitudes and origins.

When harvesting Rhizoma Musae herbs at comparable growth stages (approximately 15 cm) and harvest times, samples from different altitudes did not display consistency in lupenone and β-sitosterol content. Furthermore, the content of the two ingredients of Rhizoma Musae from different regions was different too. The difference between the lowest and highest altitude levels was up to 5-fold, indicating that the ecological environment has an important effect on the lupenone and β-sitosterol content. Therefore, the growth environment of Rhizoma Musae herbs should be chosen with great care at the appropriate locations, like Zhizhong and Medog County (Figure 4).

**Figure 5:**
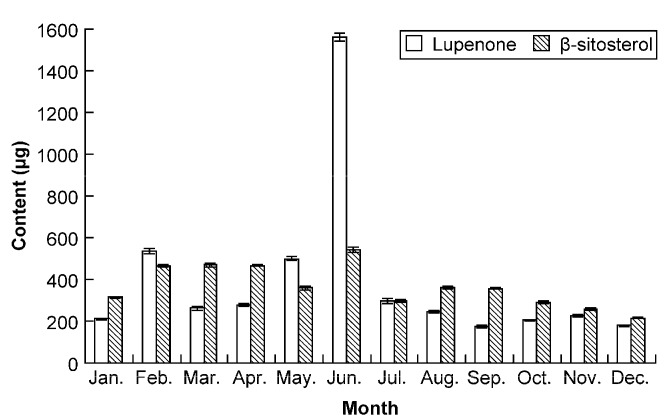
Lupenone and β-sitosterol levels in Rhizoma Musae samples collected over a 12-month period.

From Figure 5, the histogram showed that the content of lupenone and β-sitosterol in Rhizoma Musae collected from different harvest times varied widely in all the samples having comparable growth stages from the same origin. During May and June collections, the content of lupenone was relatively higher, while during collections from February to June, the content of β-sitosterol was higher than in the other months (Figure 5). The possible reason for the content difference could be that *ba-jiao* can access to adequate water supplies from January to June in China. This observation is illustrative of the degree to which the two medicinal ingredients in Rhizoma Musae are affected by season. Our results also suggest that the statement regarding herb standards described in Guizhou [2], that “Rhizoma Musae herbs can be harvested throughout the year” was rather imprecise.

**Figure 6:**
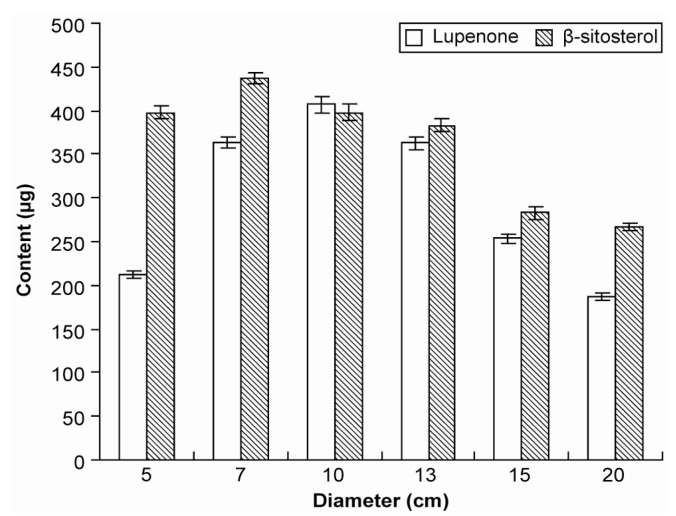
Lupenone and β-sitosterol levels in Rhizoma Musae at different growth stages.

*Ba-jiao* is a perennial herb, and when we measured the largest part of fresh Rhizoma Musae using a standard ruler, the obtained size (diameter) was used to represent the growth stage. The different growth stages (5, 7, 10, 13, 15, and 20 cm) of Rhizoma Musae collected at the same harvest time and origin displayed a range of lupenone and β-sitosterol levels. When the diameter of Rhizoma Musae was approximately 7–13 cm, lupenone content was higher. By contrast, to obtain higher levels of β-sitosterol, the best harvest diameter was approximately 5–10 cm (Figure 6). This observation indicated that the two ingredients did not increase with an extension of the growth period, but they displayed a nearly normal distribution. Therefore, Rhizoma Musae harvesting should be performed at a suitable growth stage to result in a maximum yield of lupenone and β-sitosterol isolation from Rhizoma Musae.

**Figure 7:**
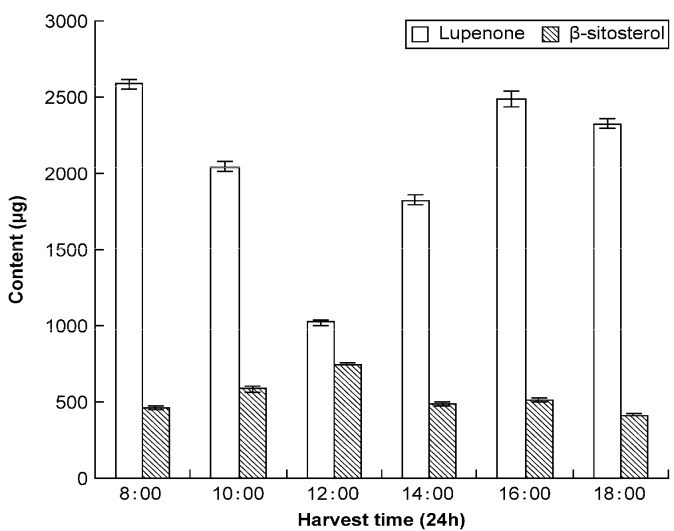
Levels of lupenone and β-sitosterol in Rhizoma Musae during different periods of the day.

The results in Table 6 show that lupenone and β-sitosterol content vary at different time periods within a single day (8:00, 10:00, 12:00, 14:00, 16:00, and 18:00). Low amounts of lupenone accumulated at 12:00–14:00, which is perhaps associated with the degree of light intensity. However β-sitosterol content displayed no major changes throughout the day (Figure 7). Therefore, Rhizoma Musae harvesting should be performed at the proper time in order to obtain optimum levels of lupenone and β-sitosterol.

### 2.5. Effects of Lupenone on Fasting Blood Glucose and HbA1c Levels in Rat

The effect of lupenone (1.78, 5.33, and 16.00 mg/kg) on FBG levels in SD rats was determined. Both positive control (rosiglitazone) and lupenone groups displayed significantly (*p* < 0.05) reduced blood glucose levels compared to the diabetic control group (Table 8). Lupenone administration also caused a significant reduction in HbA1c levels in diabetic rats at doses of 5.33 and 16.00 mg·kg^−1^·day^−1^.

Although lupenone has been reported to inhibit α-glucosidase and PTP1B enzyme, oral dosing studies in animal models have not been performed. The observations fail to explain the relationship between lupenone and diabetes. We report that lupenone action (at three different doses) affected lower blood sugar activity in a type-II diabetes animal model. Lupenone administration also caused a significant reduction in HbA1c levels. This result suggests that lupenone has potential value for development into an anti-diabetic agent.

**Table 8:** Effects of Lupenone on FBG and HbA1c levels in rats.

Group	Blood Glucose Level before Administration (mmol/L)	Blood Glucose Level after Administration (mmol/L)	HbA1c (% Hb)
Normal control	3.72 ± 0.61	3.80 ± 0.75	10.26 ± 0.93
Diabetic control	16.78 ± 3.64	17.29 ± 2.67	16.81 ± 3.00
Positive drug (1.00 mg/kg)	14.58 ± 3.11	12.45 ± 5.51 *	15.30 ± 1.21
Lupenone (1.78 mg/kg)	17.00 ± 5.12	12.47 ± 5.28 *	17.13 ± 1.31
Lupenone (5.33 mg/kg)	15.36 ± 2.17	12.07 ± 5.91 *	15.10 ± 1.52 *
Lupenone (16.00 mg/kg)	16.33 ± 3.49	10.38 ± 4.02 **	14.50 ± 1.47 **

All the values are expressed as mean ± SD; *n* = 10; * *p* < 0.05 values are significant compared to control; ** *p* < 0.01 values are significant compared to control.

## 3. Experimental Section

### 3.1. Samples, Chemicals, and Reagents

Thirty seven samples of Rhizoma Musae were identified by X.P. Wang, Department of Pharmacognosy Guiyang College of Traditional Chinese Medicine, China. All voucher specimens were deposited in the Department of Pharmacognosy, Guiyang College of Traditional Chinese Medicine, Guiyang, China. Lupenone was isolated previously from the rhizomes of Rhizoma Musae by chromatographic separation on a silica gel column, and its structure was elucidated by comparison of its spectral data (mp, MS, ^1^H-NMR) with reference standards [9]. The purity of lupenone was greater than 98%. β-Sitosterol (≥98%) was purchased from Shanghai Yuanye Bio-Technology Co., Ltd. (Shanghai, China). HPLC-grade methanol and acetonitrile were purchased from TEDIA Co. (Fairfield, OH, USA) and the deionized water was purchased from Wahaha Group Co., Ltd. (Hangzhou, China). All other analytical-grade solvents were purchased from Shanghai Zhenxing Chemical No. 1 Factory (Shanghai, China). Streptozotocin (STZ) was purchased from Sigma-Aldrich (St. Louis, MO, USA).

### 3.2. Animals

All experiments were performed on inbred 6-week-old male Sprague-Dawley (SD) rats (200 ± 20 g) obtained from Chongqing Tengxin Biological Technology Co., Ltd. (Chongqing, China; qualification number: SCXK chongqing 2007001). The colony was maintained under controlled conditions of temperature at 23 ± 2 °C, 50 (±5)% humidity and a 12-h light-dark cycle. All the animals in the study were cared for and treated humanely according to the national legislations of China, as well as local guidelines. The animal experiments were approved by the Ethics Committee for Animal Experiments of Guiyang College of TCM.

### 3.3. Instrumentation and Chromatographic Conditions

The HPLC system used was a Shimadzu LC-20AT instrument (Shimadzu Corp., Kyoto, Japan), with Diamonsil C18 columns (250 × 4.6 mm; 5 μm) (Beijing Dikma Technology Co., Ltd., Beijing, China) at a column temperature of 25 °C. The flow rate was 0.7 mL/min. A Rheodyne 7725i sampling valve (Cotati, CA, USA) equipped with a 20-µL sample loop was used for sample injection. The mobile phase was acetonitrile-methanol (50:50, v/v), using isocratic elution to obtain optimum resolution. The injection volume was 20 µL.

### 3.4. Standard Solution Preparation

Standard stock solutions of lupenone and β-sitosterol were prepared in methanol at concentrations of 402.00 and 404.00 µg/mL, respectively. Methanol was used to dilute the stock solution to the appropriate concentration for the experiment.

### 3.5. Sample Solution Preparation

Rhizoma Musae samples were crushed into powder, and dried at 50 °C to a consistent weight. The accurately weighed herbal sample (1.0 g) was placed into a 100-mL glass round-bottom flask, followed by the addition of methanol (50 mL). Next, the flask was weighed and heated under reflux in a water bath at 64 °C for 1.5 h, allowed to cool, and methanol was added to compensate for the loss of the solvent, followed by mixing. The resulting extract was subsequently filtered. Another 50 mL methanol was added, and the steps above were repeated for a second time. After the extract was filtered, the two filtrates were combined, and all successive filtrates were evaporated to dryness, with the residue dissolved in 10 mL of methanol. Finally, the extract was filtered through a 0.22-µm filter membrane (Tianjin Jinteng Experiment Equipment Co., Ltd., Tianjin, China) for subsequent analysis.

The effect of extraction method, solvent and time on yield was studied by using a monofactorial experimental design involving two extraction methods (reflux protocol and ultrasonication), five extraction solvents (methanol, ethanol, 95% ethanol, 50% methanol and 50% ethanol), and three extraction times (1-cycle, 2-cycle, 3-cycle 1.5 h).

### 3.6. Calibration Curves

Methanol stock solutions containing two analytes were prepared and diluted to appropriate concentrations for construction of calibration curves. Six concentrations of the two analytes solution were injected in triplicate, and the calibration curves were subsequently constructed by plotting the peak areas *vs.* the amount (µg/mL) of each analyte. The results are displayed in Table 1.

### 3.7. Limits of Detection and Quantification

Lupenone and β-sitosterol methanol stock solutions were serially diluted to appropriate experimental concentrations with methanol, and an aliquot of the diluted solutions was injected into HPLC for analysis. The limits of detection (LOD) and quantification (LOQ) under the present chromatographic conditions were determined at a signal-to-noise ratio (S/N) of 3 and 10, respectively. LOD and LOQ for each compound are displayed in Table 1.

### 3.8. Precision, Repeatability, Accuracy, and Stability

Intraday and interday variations were selected to determine the precision of the method. The samples were analyzed in triplicate t.i.d. for the intraday variability test, while the samples were examined in triplicate for three consecutive days for the interday variability test. The repeatability was confirmed with six parallel samples (July sample) in preparation and analysis. Stability was tested and analyzed at 0, 2, 4, 8, 16, and 24 h. Variations were expressed by relative standard deviations. The results are displayed in Table 2. The recovery test was used to evaluate the accuracy of this method. Accurate quantities of lupenone and β-sitosterol were added to approximately 1.0 g of Rhizoma Musae, extracted, and then analyzed. The average percent recovery and relative standard deviation (RSD) were calculated with the following formulas: recovery (%) = (quantity found − original quantity)/quantity spiked × 100%, and RSD (%) = (SD/mean) ×100%. The results are displayed in Table 2.

### 3.9. Effects of Lupenone on High-Fat Diet and Streptozotocin-Induced Type-II Diabetic Rats [20]

Six-week-old male rats were fed with high-fat diet (HFD; 15% cholesterol, 15% coconut oil, 15% granulated sugar, 55% laboratory animal feed). After four weeks of HFD feeding, the rats were subject to fasting overnight, and injected twice with low-dose STZ (first with 30 mg/kg, i.p.; second with 28 mg/kg, i.p. 24 h later) to induce partial insulin deficiency. Three days after STZ injection, rats with the similar degrees of hyperglycemia [*i.e.*, fasting blood glucose (FBG) levels ≥ 11.1 mmol/L] were randomly stratified into control or treatment groups (10 rats per group). The normal control and diabetic control groups both received the vehicle treatment (0.0256% Tween 80; 5.0 mL/kg), while the treatment groups were administered the drug rosiglitazone (1.00 mg/kg; 5.0 mL/kg) as a positive control or varying doses of lupenone (1.78, 5.33, and 16.00 mg/kg; 5.0 mL/kg) as suspensions in 0.0256% Tween 80 b.i.d. After 14 days of treatment, all groups were subjected to fasting overnight, and blood was subsequently drawn from the tail vein. Finally, blood glucose levels were measured using the One-touch Ultra Blood Glucose Monitoring System (Johnson & Johnson Medical (China) Ltd., Shanghai, China). HbA1c was measured on Bio-Rad D-10 system certified from National Glycohemoglobin Standardization Program (Bio-Rad, Hercules, CA, USA).

### 3.10. Statistical Analysis

All the values were expressed as mean ± SD for 10 rats from each group, and evaluated by one-way analysis of variance (ANOVA). Differences were considered to be statistically significant when *p* < 0.05 [21].

## 4. Conclusions

Lupenone possesses multiple biological activities and is widely distributed in many species of plants and herbs. However, the lack of an appropriate detection method to characterize lupenone content has hindered the development of lupenone as a medicinal product. In this study, we report a novel yet simple analytical HPLC method to isolate and characterize lupenone and β-sitosterol content in Rhizoma Musae, thereby allowing these agents to be used as reference standards for the detection of lupenone in other herbs. Concomitant characterization of lupenone and β-sitosterol in Rhizoma Musae by HPLC can be applied to the quality control of future Rhizoma Musae-derived medicines.

To optimize the yield of lupenone and β-sitosterol, we examined lupenone and β-sitosterol content in Rhizoma Musae from different regions, harvest times, sizes, and time points. Our results can be a useful guide for the efficient planting and harvesting of Rhizoma Musae.

We also demonstrated that lupenone treatment significantly lowered FBG and HbA1c levels in diabetic rats—The first report of lupenone’s hypoglycemic activity *in vivo*. Further investigations are required to support the development of lupenone as a therapeutic candidate for the management of diabetes. 
